# Injunction against the Boston Dental College

**Published:** 1869-07

**Authors:** 


*Injunction Against the Boston Dental College.* From the *Boston Herald* of June 14th we learn that Judge Ames issued an injunction on Saturday upon the officers of the Boston Dental College restraining them from granting the degree of Doctor of Dental Surgery to students of three years pupilage who have not attended two full courses of lectures, said courses to cover two full years, as understood at Harvard and other medical and dental colleges. The injunction was issued at the instigation of six members of the Board of Trustees, viz., John P. Ordway, Eben D. Jordan, Ammi Brown, I. M. Daly, Isaac Ayling and J. B. Coolidge. A hearing was to have been held on Saturday, June 19th, to see whether the injunction shall be made perpetual.

